# Clinical course and outcomes of COVID-19 patients with chronic obstructive pulmonary disease: A retrospective observational study in Wuhan, China

**DOI:** 10.1097/MD.0000000000029141

**Published:** 2022-05-13

**Authors:** Yang Bai, Liang Wen, Yulong Zhao, Jianan Li, Chen Guo, Xiaobin Zhang, Jiaming Yang, Yushu Dong, Litian Ma, Guobiao Liang, Yun Kou, Enxin Wang

**Affiliations:** a Department of Neurosurgery, General Hospital of Northern Theater Command, Shenyang, Liaoning, China; b Department of Gastroenterology, Tangdu Hospital, Fourth Military Medical University, Xi’an, Shaanxi, China; c Department of Ultrasound, General Hospital of Northern Theater Command, Shenyang, Liaoning, China; d Department of Medical Affairs, Air Force Hospital of Western Theater Command, Chengdu, Sichuan, China.

**Keywords:** clinical course, clinical outcome, COPD, COVID-19, SARS-CoV-2

## Abstract

Information about coronavirus disease 2019 (COVID-19) patients with pre-existing chronic obstructive pulmonary disease (COPD) is still lacking. The aim of this study is to describe the clinical course and the outcome of COVID-19 patients with comorbid COPD.

This retrospective study was performed at Wuhan Huoshenshan Hospital in China. Patients with a clear diagnosis of COVID-19 who had comorbid COPD (N = 78) were identified. COVID-19 patients without COPD were randomly selected and matched by age and sex to those with COPD. Clinical data were analyzed and compared between the two groups. The composite outcome was the onset of intensive care unit admission, use of mechanical ventilation, or death during hospitalization. Multivariable Cox regression analyses controlling for comorbidities were performed to explore the relationship between comorbid COPD and clinical outcome of COVID-19.

Compared to age- and sex-matched COVID-19 patients without pre-existing COPD, patients with pre-existing COPD were more likely to present with dyspnea, necessitate expectorants, sedatives, and mechanical ventilation, suggesting the existence of acute exacerbations of COPD (AECOPD). Greater proportions of patients with COPD developed respiratory failure and yielded poor clinical outcomes. However, laboratory tests did not show severer infection, over-activated inflammatory responses, and multi-organ injury in patients with COPD. Kaplan–Meier analyses showed patients with COPD exhibited longer viral clearance time in the respiratory tract. Multifactor regression analysis showed COPD was independently correlated with poor clinical outcomes.

COVID-19 patients with pre-existing COPD are more vulnerable to AECOPD and subsequent respiratory failure, which is the main culprit for unfavorable clinical outcomes. However, COPD pathophysiology itself is not associated with over-activated inflammation status seen in severe COVID-19.

## 1 Introduction

Coronavirus disease 2019 (COVID-19) caused by severe acute respiratory syndrome coronavirus-2 (SARS-CoV-2) still remains pandemic.^[1]^ Up to August 2021, World Health Organization (WHO) reported over 4.2 million death cases.^[2]^ Clinical outcomes including mortality, were worse in males, the elderly, and those with comorbidities including hypertension, diabetes, and cardiovascular disease.^[3]^ As a common senile disease, chronic obstructive pulmonary disease (COPD) is another comorbidity among patients hospitalized with COVID-19.^[4]^ A series of meta-analyses^[5–7]^ and retrospective studies^[4,8–13]^ have shown that comorbid COPD was correlated with the severity and mortality of COVID-19. Nevertheless, the clinical characteristics of COVID-19 patients with COPD and the impact of COPD on the clinical course and outcomes of the disease have not yet been well delineated.

Prior reports have indicated that COVID-19 patients with comorbid COPD were more likely to be male, with older age and more comorbidities, than those without COPD.^[8,9,11,12]^ Nevertheless, the influence of differences in these demographical features between COVID-19 patients with and without COPD has not been considered yet. To examine the impact of the pathophysiology of COPD on the clinical course of COVID-19, clinical data of COVID-19 patients with pre-existing COPD hospitalized at Wuhan Huoshenshan Hospital and age- and sex-matched COVID-19 patients without COPD randomly selected from the same cohort were compared. The association of comorbid COPD with clinical outcomes of COVID-19 was also explored *via* multivariable stepwise Cox regression.

## 2 Methods

### 2.1 Study participants

In this single-center retrospective observational study, a total of 2994 consecutive patients hospitalized in Wuhan Huoshenshan Hospital from February 5 to March 15, 2020 were screened and assessed. The inclusion criteria were:

1. patients with confirmed diagnosis of COVID-19 by detection of SARS-CoV-2 via real-time PCR according to World Health Organization interim guidelines^[14]^; and
2. patients with intact clinical data.

**Figure 1:**
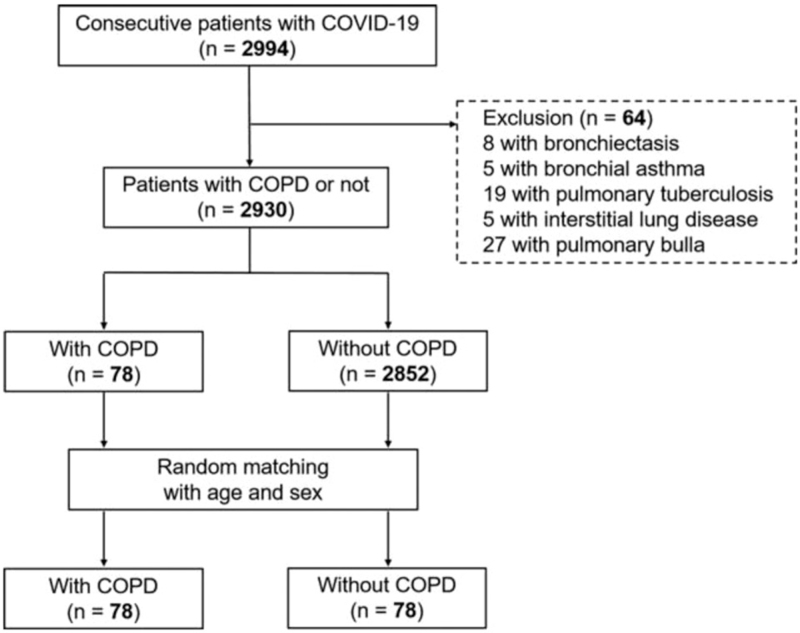
Flowchart of patient recruitment.

This tertiary hospital, located in the province of Hubei, was assigned to treat COVID-19 patients by the Chinese government during the outbreak of this pandemic. To avoid the influence of pathophysiology of other respiratory diseases, patients with pre-existing bronchiectasis, bronchial asthma, pulmonary tuberculosis, interstitial lung disease, and pulmonary bulla were excluded from this analysis (Fig. 1).

A total of 78 COVID-19 patients were diagnosed with COPD (ICD code: J44) by a physician based on prior electronic medical records or the report of the patients themselves and their family members. Compared with the overall population in the cohort, those with COPD had a significant older age (median [IQR], 70.5 [65.3**–**79.8] vs 60.0 [49.0**–**68.0]; *P* < .001) and had a significant higher proportion of male sex (71.8% vs 51.1%; *P* < .001). Prior studies has shown that both male sex and older age are independently correlated with in-hospital death in COVID-19 patients.^[15]^ Considering this, an age- (±2 years) and sex-matched patient without COPD was randomly selected for each patient in the COPD group in order to adjust for age and sex according to the method described before.^[16]^ If there were several non-COPD patients available for one patient with COPD, a match was randomly made from these available candidate patients. In total, we enrolled 156 COVID-19 patients into this study and collected their data retrospectively (Fig. 1). The present study approved by the Ethics Committee of Wuhan Huoshenshan hospital (K202101-02).

### 2.2 Data collection

All clinical data were collected from the electronic medical records by two physicians (Y Bai and L Wen), and independently checked by another two researchers (Y Zhao and J Li). Clinical data acquired herein included demographics (i.e., age, sex, and pre-existing diseases), initial symptoms (e.g., fever, myalgia, fatigue, and cough), vital signs (i.e., heart rate, blood pressure, body temperature, and respiratory rate), initial computerized tomography (CT) scan data, laboratory parameters (i.e., infection-related indices, blood routine test, coagulation function markers, myocardial impairment markers, liver function indices, kidney function indices, electrolytes, and glucose), and complications (e.g., cardiac insufficiency and respiratory failure). For imaging data, infection volume was measured using a simple CT post-processing tool, and expressed in cubic centimeters.^[17]^

In addition, clinical data concerning therapeutics were also collected. In accordance with the WHO interim guidelines,^[14]^ the patients were given individualized systematic treatments, including oxygen support, antivirals, secondary infection control, immune-modulators, and multi-organ support. Oxygen therapy was the key therapeutic approach among these treatments, which was administrated through nasal cannulas (normal or high-flow) and mechanical ventilation (non-invasive or invasive). For the treatment of COPD, anti-asthmatics included muscarinic antagonists (long-acting or short-acting), beta-2 agonists (long-acting or short-acting), inhaled corticosteroid, leukotriene receptor antagonist, phosphodiesterase-4 inhibitors, while expectorants included acetylcysteine, ambroxol, and eucalyptus enteric soft capsules.

A composite endpoint consisting of unfavorable clinical outcomes was adopted as the primary outcome, which included the occurrence of admission to intensive care unit (ICU), use of mechanical ventilation, or death. Secondary outcomes included the occurrence of virus clearance, disease progression, disease deterioration, and death. The patients were followed-up until discharge from hospital after recovery or death. The duration from symptom onset to hospital admission, virus clearance, disease progression, disease deterioration, death, or the composite endpoint was also recorded.

### 2.3 Definitions

The diagnosis of COVID-19 was based on the WHO interim guidance.^[14]^ Disease severity was determined according to the guideline for the diagnosis and management of COVID-19 (6th edition) published by the National Health Commission of China.^[18]^ Disease progression and deterioration were defined as the exacerbation of disease from non-severe types (including both mild and moderated types) to severe type and critical type, respectively. Viral clearance was considered with three consecutive negative nucleic acid results from respiratory tract specimens produced with sampling intervals more than 24 hours.

The occurrence of complications was made according to the following standards. Anemia was diagnosed with haemoglobin ≤110 g/L. The diagnosis of myocardial injury was made with the serum levels of CKMB or cardiac troponin I (cTnI) exceeding the upper limit of normal (ULN).^[19]^ Liver injury was diagnosed with the total bilirubin (TBIL) level more than twofold the ULN, or the serum levels of alanine transaminase (ALT) or aspartate aminotransferase (AST) more than threefold the ULN.^[20]^ Hypoproteinemia was determined by the serum albumin level less than 25 g/L.^[15]^ The occurrence of acute kidney injury (AKI) was confirmed by an increase in serum creatinine levels to more than 1.5 times the baseline.^[21]^ Disseminated intravascular coagulation (DIC) and sepsis-induced coagulopathy (SIC) were determined by the International Society of Thrombosis and Haemostasis scoring systems.^[22,23]^ Cardiac insufficiency was reported if the serum level of brain natriuretic peptide (BNP) exceeded the normal range, together with the presence of dyspnea, orthopnea, and edema of lower-extremity.^[24]^ Hepatic failure was diagnosed in accordance with clinical practical guidelines on the management of acute liver failure released by European Association for the Study of the Liver.^[25]^ Acute respiratory distress syndrome (ARDS) was defined according to the Berlin definition.^[26]^ The diagnosis of respiratory failure was made by an arterial partial pressure of oxygen of <60 mm Hg. Multiple organ dysfunction syndrome (MODS) and shock were defined based on the multiple organ dysfunction score^[27]^ and the 2016 Third International Consensus Definition for Sepsis and Septic Shock,^[28]^ respectively.

### 2.4 Statistical analysis

For variables with missing data, no imputation was performed. Data is expressed as percentages (%) for enumeration data or medians (interquartile ranges [IQRs]) for quantitative data. Intergroup comparisons between COPD group and non-COPD group were performed using the χ^2^ test (or Fisher's exact test) for enumeration data or the Mann–Whitney *U* non-parametric test for quantitative data. Cumulative survival curves were plotted using the Kaplan–Meier method together with the log-rank test.

Cox regression analyses were performed to determine if pre-existing COPD was an independent predictor for the primary outcome. Since the aim of this study was to document the relationship between demographic variables and the clinical outcome of COVID-19, age, sex and pre-existing diseases were included in the univariable analysis. Then, three variables, that is, age, coronary heart disease (CHD), and COPD, were incorporated into the multivariable model based on the results of univariable analyses and their clinical merits mentioned in previous findings.^[15,29–31]^ Atrial fibrillation and chronic kidney disease were excluded from the multifactor model owing to the small number of events. All statistical analyses were made using SPSS software (version 22.0, IBM Corp, NY). *P* < .05 were considered statistically significant.

## 3 Results

### 3.1 Identification of COVID-19 patients with COPD

A total of 2994 consecutive hospitalized patients with a clear diagnosis of COVID-19 were screened from the medical record system. Among them, 8 patients with bronchiectasis, 5 patients with bronchial asthma, 19 patients with pulmonary tuberculosis, 5 patients with interstitial lung disease, and 27 patients with pulmonary bulla (diagnosed in previous health examinations or on admission) were excluded. Finally, 78 patients with pre-existing COPD were included (Fig. 1). Of these 78 patients, 6 cases were at acute attack phase and 2 cases had developed pulmonary heart disease. The median interval from the diagnosis of COPD to admission due to COVID-19 was 10.0 years (IQR, 7.3–20.0 years).

### 3.2 Demographic and clinical features

Demographic and clinical features of COVID-19 patients in the COPD group and the non-COPD group were indicated in Table 1. After matching, no difference could be seen in the prevalence of pre-existing diseases, such as hypertension and CHD (*P* > .05) between the COPD group and the non-COPD group. Unexpectedly, no significant difference in disease severity was observed in the two groups (62.8% in COPD group vs 61.5% in non-COPD group; *P* = .868). Concerning initial symptoms and signs, dyspnea occurred more frequently in those with than without COPD (59.0% vs 37.2%; *P* < .01). For vital signs, there was a trend towards increased initial (87.0 [79.8–98.0] vs 82.5 [77.8–94.0] beats per min; *P* = .081) and peak (107.5 [98.0–115.0] vs 102.0 [98.0–112.0] beats per min; *P* = .087) HR during hospitalization in patients with COPD. Importantly, no significant difference in pulmonary infection volume and proportion (both *P* > .05) was seen between these groups.

**Table 1:** Demographic and clinical features of matched COVID-19 patients with and without COPD.

	Patients with COPD	Patients without COPD	
	(n = 78)	(n = 78)	*P* ^∗^
Age, y	70.5 (64.8–80.5)	70.5 (64.8–80.5)	.992
Male, No. (%)	55 (70.5)	55 (70.5)	1.000
Symptom onset to admission, d	29.0 (14.5–45.0)	22.0 (15.8–40.0)	.234
Comorbidity, No. (%)			
Hypertension	31 (39.7)	28 (35.9)	.620
Diabetes	6 (7.7)	14 (17.9)	.055
CHD	15 (19.2)	15 (19.2)	1.000
AF	5 (6.4)	1 (1.3)	.210^∗^
CVD	5 (6.4)	5 (6.4)	1.000
CKD	2 (2.6)	1 (1.3)	1.000^∗^
Malignancy	4 (5.1)	6 (7.7)	.513^∗^
Disease classification, No. (%)			
Non-severe group	49 (62.8)	48 (61.5)	.868
Severe group	29 (37.2)	30 (38.5)	
Severe type	22 (28.2)	29 (37.2)	
Critical type	7 (9.0)	1 (1.3)	
Initial symptoms and signs, No. (%)			
Asymptomatic	4 (5.1)	6 (7.7)	.513
Fever	51 (65.4)	52 (66.7)	.866
Max. Temp, °C	38.3 (37.7–38.9)	38.5 (38–38.8)	.233
Chill	13 (16.7)	14 (17.9)	.832
Myalgia	28 (35.9)	18 (23.1)	.079
Fatigue	42 (53.8)	32 (41.0)	.109
Respiratory symptoms
Dry cough	60 (76.9)	52 (66.7)	.155
Productive cough	19 (24.4)	15 (19.2)	.438
Dyspnea	46 (59.0)	29 (37.2)	**.006**
Nasal congestion	1 (1.3)	1 (1.3)	1.000^∗^
Rhinorrhoea	1 (1.3)	2 (2.6)	1.000^∗^
Pharyngalgia	5 (6.4)	2 (2.6)	.442^∗^
Chest tightness	19 (24.4)	15 (19.2)	.438
Chest pain	1 (1.3)	1 (1.3)	1.000^∗^
Gastrointestinal symptoms			
Anorexia	6 (7.7)	9 (11.5)	.415
Nausea	0 (0)	2 (2.6)	.497^∗^
Vomiting	1 (1.3)	1 (1.3)	1.000^∗^
Diarrhea	3 (3.8)	5 (6.4)	.719^∗^
Abdominal distention	1 (1.3)	1 (1.3)	1.000^∗^
Abdominal pain	1 (1.3)	1 (1.3)	1.000^∗^
Vital signs, median (IQR)			
HR, beats per min	87.0 (79.8–98.0)	82.5 (77.8–94.0)	.081
SBP, mmHg	132.0 (122.0–140.0)	130.0 (118.8–137.3)	.276
DBP, mmHg	82.0 (72.0–88.0)	78.0 (71.0–86.0)	.272
RR, breaths per min	20.0 (20.0–20.0)	20.0 (20.0–21.0)	.253
Temp, °C	36.5 (36.5–36.6)	36.5 (36.2–36.8)	.206
Max. HR, beats per min	107.5 (98.0–115.0)	102.0 (98.0–112.0)	.087
Max. RR, breaths per min	23.0 (22.0–27.0)	22.5 (22.0–26.0)	.216
Max. Temp, °C	37.1 (36.9–37.5)	37.1 (36.9–37.4)	.811
Radiologic characteristics, median (IQR)			
Infection volume, cm^3^	230.7 (51.8–562.6)	353.0 (94.3–628.6)	.355
Infection proportion, %	5.8 (1.4–21.8)	11.6 (2.2–16.9)	.426

∗ Compared by Fisher's exact test. AF = atrial fibrillation, CHD = coronary heart disease, CKD = chronic kidney disease, COPD = chronic obstructive pulmonary disease, CVD = cerebrovascular disease, DBP = diastolic blood pressure, HR = heart rate, Max. = maximum, RR = respiratory rate, SBP = systolic blood pressure, Temp. = temperature.

### 3.3 Laboratory findings

No significant difference in initial laboratory parameters (Table 2), including infection-related indicators, blood routine test, coagulation function biomarkers, myocardial damage markers, liver and kidney function indices, electrolytes, and glucose (all *P* > .05), were observed between patients with COPD and without COPD. In addition to initial laboratory values, the maximus or minimum values of some indicators during hospitalization of utmost importance for the diagnosis of complications were also collected and analyzed. Those with COPD vs those without COPD only had a higher level of peak IL-6 (5.6 [2.6–29.9] vs 3.0 [1.5–7.7] pg/mL; *P* = .017). No significant difference in peak or lowest value of other indicators reflecting infection or multiple organ injury (all *P* > .05) was observed between the two groups.

**Table 2:** Laboratory tests of matched COVID-19 patients with and without COPD.

	Patients with COPD	Patients without COPD	
	(n = 78)	(n = 78)	*P*
Initial laboratory examination (normal range), median (IQR)			
Infection-related indices			
IL-6, pg/mL (<7)	4.3 (1.8–13.3)	2.9 (1.5–7.7)	.249
CRP, mg/L (0–4)	5.6 (1.4–30.4)	4.9 (1.3–28.6)	.518
PCT, ng/mL (0–0.05)	0.05 (0.04–0.11)	0.05 (0.03–0.07)	.312
WBC, ×10^9^/L (3.5–9.5)	6.3 (4.7–7.7)	5.7 (4.6–7.1)	.252
NEU, ×10^9^/L (1.8–6.3)	3.9 (2.9–5.3)	3.7 (2.8–5.1)	.584
LYM, ×10^9^/L (1.1–3.2)	1.3 (0.8–2.0)	1.2 (0.8–1.6)	.227
NLR, (1.8–5.7)	2.7 (1.9–5.0)	2.7 (2.1–5.6)	.625
Blood routine test			
RBC, ×10^12^/L (3.8–5.1)	3.9 (3.5–4.3)	3.9 (3.5–4.3)	.972
Hb, g/L (115–150)	124.0 (109.0–134.0)	121.0 (104.8–131.3)	.329
PLT, ×10^9^/L (125–350)	221.0 (164.0–272.0)	211.0 (161.8–262.0)	.654
Coagulation function test			
FIB, g/L (1.8–3.5)	3.1 (2.8–3.7)	3.2 (2.8–3.6)	.749
APTT, s (21–37)	29.0 (26.7–30.6)	29.3 (27.15–32.5)	.426
PT, s (9.2–15)	13.2 (12.3–14.0)	13.1 (12.4–14.4)	.571
TT, s (14–21)	15.4 (14.5–16.5)	15.5 (14.6–16.3)	.478
INR, (0.8–1.25)	1.1 (1.0–1.2)	1.1 (1.1–1.2)	.772
D-Dimer, mg/L (0–0.55)	1.0 (0.4–2.0)	0.9 (0.5–1.5)	.411
PTA, (70–125)	93.4 (89.3–98.2)	94.1 (88.1–98.0)	.804
Myocardial injury markers			
CKMB, IU/L (0–24)	9.9 (7.6–12.4)	8.9 (7.5–12.2)	.531
LDH, IU/L (120–250)	202.1 (165.2–246.5)	182.8 (155.5–234.9)	.163
α-HBDH, IU/L (72–182)	168.9 (134.3–198.4)	154.8 (127.1–194.2)	.273
MYO, ng/mL (0–65)	11.2 (6.3–22.4)	8.9 (5.3–20.5)	.354
cTnI, ng/mL (0–0.04)	0.01 (0.01–0.02)	0.01 (0.01–0.02)	.510
BNP, pg/mL (0–100)	25.9 (0.0–109.5)	36.7 (0.0–116.3)	.749
Liver function indices			
ALT, IU/L (7–40)	18.0 (11.9–27.5)	21.4 (13.1–35.9)	.221
AST, IU/L (7–45)	19.2 (15.6–26.1)	20.8 (16.2–25.8)	.764
TB, g/L (20–30)	63.6 (58.6–67.7)	60.2 (57.4–65.5)	.064
ALB, g/L (40–55)	35.6 (32.3–38.4)	35.3 (31.9–37.4)	.400
TBIL, μmol/L (0–21)	10.0 (7.4–12.7)	10.1 (8.2–13.7)	.585
DBIL, μmol/L (0–8)	3.7 (2.7–4.7)	3.6 (2.6–4.9)	.800
γ-GT, IU/L (7–45)	31.2 (21.6–46.3)	25.0 (18.2–40.8)	.178
Kidney function indices			
BUN, mmol/L (3.1–8.8)	5.0 (4.0–6.5)	5.0 (3.9–6.0)	.346
Cr, μmol/L (41–81)	69.0 (60.7–81.3)	69.8 (57.6–81.3)	.826
CysC, mg/L (22–29)	1.1 (0.9–1.3)	1.1 (0.9–1.2)	.401
Electrolytes and glucose			
CO_2_, mmol/L (22–29)	25.0 (22.8–27.0)	24.7 (23.0–25.9)	.309
Na^+^, mmol/L (137–147)	140.9 (138.7–143.0)	140.6 (138.8–142.4)	.596
K^+^, mmol/L (3.5–5.3)	4.3 (4.0–4.6)	4.2 (3.9–4.6)	.393
Ca^2+^, mmol/L (211–252)	2.1 (2.0–2.2)	2.1 (2.0–2.2)	.072
Cl^-^, mmol/L (99–110)	105.1 (102.2–107.2)	105.5 (103.8–107.4)	.283
Glu, mmol/L (3.9–6.1)	4.9 (4.5–5.7)	5.0 (4.6–5.8)	.371
Peak/lowest value during hospitalization (normal range), median (IQR)			
Max. IL-6, pg/mL (<7)	5.6 (2.6–29.9)	3.0 (1.5–7.7)	**.017**
Max. CRP, mg/L (0–4)	10.5 (2.4–57.8)	5.6 (1.8–37.6)	.294
Max. PCT, ng/mL (0–0.05)	0.05 (0.04–0.16)	0.05 (0.03–0.10)	.208
Min. Hb, g/L (115–150)	118.0 (102.0–130.0)	114.0 (99.5–127.5)	.285
Max. WBC, ×10^9^/L (3.5–9.5)	7.2 (5.6–10.1)	6.7 (4.9–8.4)	.041
Min. PLT, ×10^9^/L (125–350)	198.0 (130.0–234.5)	184.0 (150.0–235.5)	.768
Min. FIB, g/L (1.8–3.5)	2.9 (2.5–3.2)	2.9 (2.5–3.3)	.583
Max. PT, s (9.2–15)	13.4 (12.6–14.7)	13.2 (12.6–14.5)	.690
Max. TT, s (14–21)	15.8 (14.9–17.1)	15.8 (15.1–16.9)	.943
Max. INR, (0.8–1.25)	1.1 (1.1–1.2)	1.1 (1.1–1.2)	.539
Max. D-Dimer, mg/L (0–0.55)	1.2 (0.5–2.7)	1.0 (0.5–2.3)	.512
Min. PTA, (70–125)	92.2 (85.9–96.9)	94.1 (88.0–97.6)	.433
Max. CKMB, IU/L (0–24)	11.4 (8.4–16.3)	10.1 (7.7–14.7)	.119
Max. cTnI, ng/mL (0–0.04)	0.01 (0.01–0.02)	0.01 (0.01–0.02)	.510
Max. BNP, pg/mL (0–100)	46.9 (10.3–174.2)	48.8 (0.0–198.4)	.992
Max. ALT, IU/L (7–40)	23.9 (13.9–42.6)	23.8 (14.8–43.6)	.807
Max. AST, IU/L (7–45)	23.1 (17.1–36.4)	24.0 (18.1–30.9)	.958
Min. ALB, g/L (40–55)	34.5 (29.6–38.3)	34.5 (31.5–36.8)	.777
Max. TBIL, μmol/L (0–21)	10.2 (8.0–14.2)	10.4 (8.5–13.7)	.800
Max. Cr, μmol/L (41–81)	73.6 (61.9–84.9)	72.8 (61.6–83.8)	.757

ALB = albumin, ALT = alanine transaminase, APTT = activated partial thromboplastin time, AST = aspartate transaminase, BNP = brain natriuretic peptide, BUN = blood urea nitrogen, CKMB = creative kinase MB, COPD = chronic pulmonary obstructive disease, Cr = creatinine, CRP = C-reactive protein, cTnI = cardiac tropinin I, CysC = cystatin C, DBIL = direct bilirubin, FIB = fibrinogen, Glu = glucose, Hb = hemoglobin, IL-6 = interleukin-6, INR = international normalized ratio, LDH = lactate dehydrogenase, LYM = lymphocyte, MYO = myoglobin, NEU = neutrophil, NLR = neutrophil-to-lymphocyte ratio, PCT = procaicltonin, PLT = blood platelet, PT = prothrombin time, PTA = prothrombin activity, RBC = red blood cell, TBIL = total bilirubin, TT = thrombin time, WBC = white blood cell.

### 3.4 Complications and treatments

Compared with those without COPD, patients with COPD had a significant higher likelihood of progressing to respiratory failure (19.2% vs 5.1%; *P* < .01), and a marginally significant higher likelihood of developing cardiac insufficiency (10.3% vs 2.6%; *P* = .050) (Table 3). With regard to treatments, patients with pre-existing COPD more likely required mechanical ventilation (16.7% vs 6.4%; *P* < .05) than their non-COPD counterparts. For symptomatic treatment, these patients were more often treated with sedatives and analgesics (10.3% vs 2.6%; *P* = .050), beta-blockers (32.1% vs 17.9%; *P* = .040) and antianginal drugs (25.6% vs 11.5%; *P* = .020) for treating discomfort, tachycardia and symptoms of myocardial ischemia, respectively. Although more likely representing dyspnea upon admission, the use of anti-asthmatic agents was not more frequently used in patients with COPD. Instead, greater proportions of COPD patients required expectorants than their non-COPD counterparts (Table 4).

**Table 3:** Complications and outcomes of matched COVID-19 patients with and without COPD.

	Patients with COPD	Patients without COPD	
	(n = 78)	(n = 78)	*P* ^∗^
Complications, No. (%)			
Cardiac injury	13 (16.7)	7 (9.0)	.151
Cardiac insufficiency	8 (10.3)	2 (2.6)	**.050**
Acute liver injury	7 (9.0)	2 (2.6)	.167^∗^
Hypoproteinemia	7 (9.0)	1 (1.3)	.063^∗^
Anemia	35 (44.9)	44 (56.4)	.106
AKI	16 (20.5)	12 (15.4)	.404
SIC	15 (19.2)	14 (17.9)	.837
DIC	5 (6.4)	3 (3.8)	.719^∗^
ARDS	6 (7.7)	2 (2.6)	.276^∗^
Respiratory failure	15 (19.2)	4 (5.1)	**.007**
Hepatic failure	1 (1.3)	1 (1.3)	1.000^∗^
Kidney failure	3 (3.8)	1 (1.3)	.620^∗^
MODS	4 (5.1)	3 (3.8)	1.000^∗^
Septic shock	5 (6.4)	1 (1.3)	.210^∗^
Outcomes, No. (%)			
Length of stay, median (IQR), d	13.5 (8.0–18.3)	14.0 (8.0–21.3)	.740
Disease progression	45 (57.7)	36 (46.2)	.149
Disease deterioration	20 (25.6)	9 (11.5)	**.024**
Composite endpoint	16 (20.5)	5 (6.4)	**.010**
Death	8 (10.3)	2 (2.6)	**.050**
Onset of symptom to, median (IQR), d			
Disease progression	31.5 (15.0–49.3)	31.0 (18.0–44.0)	.975
Disease deterioration	41.0 (21.0–54.0)	39.0 (30.0–51.0)	.734
Discharge	50.0 (31.8–59.3)	40.0 (32.0–53.3)	.166
Composite endpoint	46.0 (26.0–55.3)	40.0 (31.0–52.3)	.861

∗ Compared by Fisher's exact test. AKI = acute kidney injury, ARDS = acute respiratory distress syndrome, COPD = chronic pulmonary obstructive disease, DIC = disseminated intravascular coagulation, GIB = gastrointestinal bleeding, MODS = multiple organ dysfunction syndrome, SIC = sepsis-induced coagulopathy.

**Table 4:** Treatment of matched COVID-19 patients with and without COPD.

	Patients with COPD	Patients without COPD	
Treatments, No. (%)	(n = 78)	(n = 78)	*P* ^∗^
Oxygen therapy			
Oxygen inhalation	70 (89.7)	72 (92.3)	.575
High-flow nasal cannula	9 (11.5)	4 (5.1)	.148
Mechanical ventilation	13 (16.7)	5 (6.4)	**.045**
Non-invasive	11 (14.1)	3 (3.8)	
Invasive	7 (9.0)	2 (2.6)	
Renal replacement therapy	4 (5.1)	1 (1.3)	.367^∗^
Bronchoalveolar lavage	1 (1.3)	1 (1.3)	1.000^∗^
Antivirals	43 (55.1)	39 (50.0)	.521
Umidenovir	38 (48.7)	33 (42.3)	
Oseltamivir	5 (6.4)	9 (11.5)	
Ribavirin	4 (5.1)	3 (3.8)	
Interferon	4 (5.1)	12 (15.4)	
Chloroquine	3 (3.8)	2 (2.6)	
Antibacterials			
First and second-line antibacterials	37 (47.4)	33 (42.3)	.520
Quinolones	32 (41.0)	30 (38.5)	
Cephalosporins	5 (6.4)	8 (10.3)	
Macrolides	3 (3.8)	0 (0)	
Third-line antibacterials	19 (24.4)	12 (15.4)	.160
Cephalosporins	0 (0)	1 (1.3)	
β-lactamase inhibitors	17 (21.8)	10 (12.8)	
Carbapenems	8 (10.3)	3 (3.8)	
Vancomycin	0 (0)	1 (1.3)	
Tigecycline	1 (1.3)	0 (0)	
Linezolid	5 (6.4)	4 (5.1)	
Antifungals	6 (7.7)	5 (6.4)	.754
Immunomodulators			
Glucocorticoids	29 (37.2)	21 (26.9)	.170
Immunoglobulin	5 (6.4)	7 (9.0)	.548
Thymosin	23 (29.5)	23 (29.5)	1.000
Tocilizumab	8 (10.3)	4 (5.1)	.229
Convalescent plasma	6 (7.7)	8 (10.3)	.575
Mesenchymal stem cell therapy	6 (7.7)	3 (3.8)	.495^∗^
Drugs for cardiovascular disorders			
Cardiotonic drugs	8 (10.3)	5 (6.4)	.385
Beta-blockers	25 (32.1)	14 (17.9)	**.042**
Amiodarone	4 (5.1)	0 (0)	.120^∗^
Antianginal drugs	20 (25.6)	9 (11.5)	**.024**
Creatine phosphate	2 (2.6)	2 (2.6)	1.000^∗^
Antilipemic agents	9 (11.5)	12 (15.4)	.482
Anticoagulants	12 (15.4)	16 (20.5)	.404
Drugs for gastrointestinal disorders			
Acid inhibitors	33 (42.3)	27 (34.6)	.323
Laxatives	15 (19.2)	23 (29.5)	0.136
Antidiarrheics	6 (7.7)	7 (9.0)	1.000
Gastrointestinal stimulants	13 (16.7)	7 (9.0)	.151
Probiotics	21 (26.9)	28 (35.9)	.227
Hepatic protectants	7 (9.0)	13 (16.7)	.151
Drugs for respiratory disorders			
Anti-asthmatics	43 (55.1)	34 (43.6)	.150
Expectorants	46 (59.0)	28 (35.9)	**.004**
NSAIDs	15 (19.2)	12 (15.4)	.525
Sedatives & analgesics	8 (10.3)	2 (2.6)	**.050**

∗ Compared by Fisher's exact test. COPD = chronic pulmonary obstructive disease, NSAID = nonsteroidal anti-inflammatory drug.

### 3.5 Outcomes

**Figure 2:**
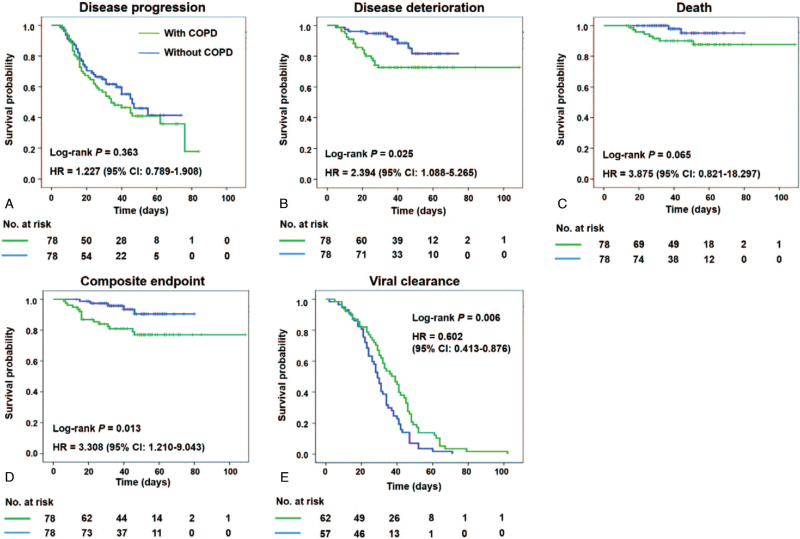
Kaplan–Meier plots for different clinical outcomes in patients with and without chronic obstructive pulmonary disease. The figure displays the Kaplan–Meier survival plots according to disease progression (A), disease deterioration (B), death (C), composite endpoint (D), and viral clearance (E). COPD = chronic obstructive pulmonary disease, HR = hazard ratio.

As shown in Table 3, patients with COPD more likely experienced disease deterioration (25.6% vs 11.5%; *P* = .024) instead of disease progression (57.7% vs 46.2%; *P* = .149). Despite no significant difference seen in the length of stay (*P* = .740) between the two groups, those with COPD had worse clinical outcomes (20.5% vs 6.4%; *P* = .010), with marginally significant higher rate of death (10.3% vs 2.6%; *P* = .050). Kaplan–Meier survival curve also indicated that although pre-existing COPD did not influence the risk of disease progression (hazard ratio [HR], 1.227; 95% CI, 0.789–1.908; *P* = .363), it increased the risk of disease deterioration by 139.4% (HR, 2.394; 95% CI, 1.088–5.265; *P* = .025) and the risk of unfavorable clinical outcomes by 230.8% (HR, 3.308; 95% CI, 1.210–9.043; *P* = .013) in patients with COVID-19. In addition, there was a trending increase in the risk of mortality in those with COPD compared with their non-COPD counterparts (HR, 3.875; 95% CI, 0.821–18.297; *P* = .065) (Fig. 2A–D). Finally, viral clearance was determined *via* PCR test for SARS-COV-2 in upper respiratory tract specimens. Surprisingly, we observed that the median time from symptom onset to negative PCR result was significantly longer in COVID-19 patients with COPD compared to their non-COPD counterparts (39.0 [30.4–47.6] vs 29.0 [26.0–32.0] days; *P* = .006) (Fig. 2E).

Initial univariable Cox regression analyses of demographic variables identified COPD as a risk factor (HR, 3.768; 95% CI, 1.306–10.872; *P* = .014) for adverse clinical outcomes. Subsequently, a multivariable model conducted with a forward stepwise approach, which incorporated age, COPD, and CHD, showed that COPD was an independent predictor (HR, 3.915; 95% CI, 1.337–11.463; *P* = .013) for unfavorable outcome of COVID-19 (Table 5).

**Table 5:** Cox regression analyses of risk factors for outcome for composite endpoint of matched COVID-19 patients with and without COPD.

	Univariable analysis		Multivariable analysis	
Variable	HR (95% CI)	*P*	HR (95% CI)	*P*
Age, y	1.042 (1.000–1.085)	.048	1.044 (1.001–1.088)	.043
Male	1.789 (0.567–5.652)	.321		
Hypertension	1.994 (0.790–5.003)	.144		
Diabetes	2.500 (0.801–7.806)	.115		
CHD	2.435 (0.885–6.699)	.085	–	–
CVD	1.750 (0.129–23.703)	.674		
COPD	3.768 (1.306–10.872)	.014	3.915 (1.337–11.463)	.013
Malignancy	1.671 (0.330–8.467)	.535		

The multivariable model contains age, CHD, and COPD. CHD = coronary heart disease, CKD = chronic kidney disease, COPD = chronic obstructive pulmonary disease, CVD = cerebrovascular disease, HR = hazard ratio.

## 4 Discussion

This study touched the clinical characteristics of COVID-19 patients with COPD in comparison to sex-and age-matched COVID-19 patients without COPD. COPD was identified as an independent risk factor of unfavorable outcomes of COVID-19. Among COVID-19 patients hospitalized in Wuhan Huoshenshan Hospital from February 5 to March 15, 2020, the prevalence of COPD was 2.6% (78/2994). In comparison with their non-COPD counterparts, patients with COPD more likely suffered respiratory failure and cardiac insufficiency, had disease deterioration, and yielded worse clinical outcomes. However, those with COPD did not show a propensity for over-activated inflammation status and subsequent multi-organ injury.

Dyspnea is a common symptom in COVID-19 patients, especially in severe cases.^[32]^ It is caused by the damage of lung tissue and exudation of pulmonary odema fluid owing to the over-activated immunoreaction during SARS-CoV-2 infection.^[33]^ Apart from pulmonary causes, the nervous system is also implicated in the development of dyspnea. The dyspnea neurocircuitry includes proprioceptive and broncho-pulmonary afferents and centrally generated inspiratory motor output, with the sensorimotor cortex, limbic system, and brainstem respiratory center involved.^[34,35]^ In our prior study, we have noticed that a lower proportion of COVID-19 patients with pre-existing cerebrovascular disease (CVD) presented with dyspnea at disease onset compared with those without CVD, which possibly resulted from the dysfunction of brain areas involved in dyspnea modulation owing to ischemic or hemorrhagic stroke.^[16]^ However, for COPD, pulmonary causes instead of neural mechanisms play a key role in dyspnea. COPD pathophysiology is characterized by chronic bronchitis and emphysema. The former leads to airway wall thickness, mucus hypersecretion, and narrowed bronchioles, while the latter induces the destruction of alveoli structure.^[36]^ Herein, we observed that a higher portion of COVID-19 patients with COPD presented with dyspnea at disease onset compared with their non-COPD counterparts. One reason is that a small proportion of the 78 COPD patients were at acute attack phase. Thus, the frequently seen dyspnea in this population may reflect COPD pathophysiology itself. Another explanation is that COPD pathophysiology and COVID-19 pathophysiology may exert synergistic effects that lead to pulmonary over-inflammation and functional collapse, which eventually lead to dyspnea and respiratory failure.

In the present study, we observed that although COVID-19 patients with COPD did not show a propensity for disease progression, COPD conferred a higher risk for disease deterioration and unfavorable clinical outcomes, which was consistent with previous studies.^[8,9,11,12]^ COPD usually occurs in later life with high prevalence of associated comorbidities. In addition, COPD itself was characterized by lung dysfunction and immune dysregulation of the airways. All these features were considered to account for worse outcomes in COVID-19 patients with pre-existing COPD.^[37–39]^ Herein, we observed that those with COPD more likely presented dyspnea, developed respiratory failure and cardiac insufficiency, and necessitated more expectorants, anti-bradycardia drugs, and mechanical ventilation than their non-COPD counterparts, suggesting the occurrence of acute exacerbation of COPD (AECOPD) in these patients. Viral or bacterial infection is the major etiological trigger for AECOPD. This kind of AECOPD, characterized by more marked impairment in pulmonary function, was severer than those with non-infectious causes (such as pulmonary embolism, smoking, and heart failure).^[40]^ Considering these, we postulated that COVID-19-triggered AECOPD and subsequent respiratory failure were the main culprit for mortality among these patients.

Cell entry of SARS-CoV-2 through angiotensin-converting enzyme 2 (ACE2),^[41]^ causes limited damage to the lung and other target organs in most cases owing to virus clearance from the respiratory system *via* adaptive immune reactions.^[42]^ However, aberrant uncontrolled response, also known as “cytokine storm,” could lead to ARDS, MODS, and even death in severe cases.^[43,44]^ Herein, clinical evidence from imaging data, laboratory tests, complications, and therapeutics did not reveal the existence of severer lung infection, cytokine storm and subsequent multi-organ injury in COVID-19 patients with COPD. This seems to contradict with prior studies by Alberca et al and He et al concluding that COVID-19 patients with COPD had severer infection and inflammatory responses.^[8,11]^ These authors believed that the up-regulation of ACE2 in COVID-19 patients with COPD was esteemed to confer a higher level of COVID-19 severity.^[45]^ However, this hypothesis was challenged by the fact that the elderly, a high-risk population for severe COVID-19, exhibited a lower level of ACE2 in the lungs.^[38,46]^ Additionally, the relatively lower expression of ACE2 within the bronchial epithelium compared with the nasal epithelium could not account for predominantly small airways pathology of COVID-19.^[47]^ In light of these, the up-regulated expression of ACE2 in patients with COPD may not be enough to explain the increased disease severity.

The possible explanation for this discrepancy is that our study design differs from those of prior studies by Alberca et al and He et al.^[8,11]^ A matching method was used in the present study to control the impact of discrepancy in demographical features on COVID-19, since the objective of our study was to examine the influence of the pathophysiology of COPD on the clinical course of COVID-19. Apart from the risk conferred by age and sex, COPD itself was correlated with worse clinical outcomes of COVID-19. Older age has been demonstrated to be an independent risk factor of adverse outcomes in patients with COVID-19.^[15]^ Apart from age-dependent impairment in adaptive immune function,^[48]^ age-related comorbidities (hypertension, diabetes, CHD, etc) reflect a state of endothelial dysfunction, rendering patients more vulnerable to multi-organ hemorrhagic and ischemic complications, especially in the lungs, kidney, brain, and heart, in the end-stage of COVID-19. Considering these, we suggest that the severer infection and immune response reported by Alberca et al and He et al was owing to the older age and more comorbidities in COVID-19 patients with COPD. COPD pathophysiology itself confers increased risk of unfavorable clinical outcomes in patients with COVID-19 mainly by dysregulation of the anti-viral immune response, and their susceptibility to virus-induced exacerbations and subsequent respiratory failure.

Our study has several potential limitations. First, it was a single-center based study with a small number of cases, rendering it difficult to assess various risk factors accurately *via* multivariable regression model. Second, the findings could not be generalized to other regions around the world with differing epidemiological features, since all study participants were from the epicenter Wuhan during the early time of disease outbreak. Third, the available data failed to deal with the heterogeneity of COPD, including disease severity and exacerbation frequency, and further studies with a larger cohort on a nationwide basis should incorporate these additional susceptibility factors. Fourth, long-term follow-up of this subpopulation with COVID-19 are urgently necessitated. Despite these shortcomings, these data will provide guidance for physicians to gain a better understanding of the panorama of this disease and are beneficial to the treatment of patients with pre-existing COPD who are at high risk for severe COVID-19.

## 5 Conclusions

This study demonstrates that compared to age- and sex-matched patients without COPD, those with COPD more likely presented acute exacerbations of COPD, developed respiratory failure, and yielded poor clinical outcomes. It is also worth noting that over-activated inflammatory responses as seen in severe COVID-19 cases may not be related to the poor outcomes of COVID-19 patients with COPD.

## Acknowledgments

We sincerely thank all patients who were involved in this study and their families, as well as all medical workers and civilians for their dedication and sacrifice in the fight against the virus.

## Author contributions

G Liang, Y Kou, and E Wang conceived the idea, designed, and supervised the study, and had full access to all the data and took responsibility for the integrity of the data. Y Bai, L Wen, and Y Zhao analyzed data and performed statistical analysis. All the authors reviewed and approved the final version of the manuscript.

**Conceptualization:** G Liang, Y Kou, and E Wang.

**Data curation:** Y Bai, L Ma, J Li, X Zhang, C Guo, and J Yang.

**Formal analysis:** Y Bai, L Wen, and Y Zhao.

**Methodology:** G Liang, Y Kou, and Y Dong.

**Project administration:** G Liang.

**Resources:** G Liang and L Ma.

**Supervision:** G Liang, Y Kou, and E Wang.

**Validation:** G Liang and Y Kou.

**Writing – original draft:** Y Bai.

**Writing – review & editing:** Y Bai and E Wang.

## References

[1] Lu R Zhao X Li J. Genomic characterisation and epidemiology of 2019 novel coronavirus: implications for virus origins and receptor binding. Lancet (London, England) 2020;395:565–74. 10.1016/S0140-6736(20)30251-8 PMC7159086 32007145

[2] Jang SH. Somatotopic arrangement and location of the corticospinal tract in the brainstem of the human brain. Yonsei Med J 2011;52:553–7. 21623594 10.3349/ymj.2011.52.4.553 PMC3104450

[3] Wu Z McGoogan JM. Characteristics of and important lessons from the coronavirus disease 2019 (COVID-19) outbreak in China: summary of a report of 72 314 cases from the Chinese Center for Disease Control and Prevention. JAMA 2020;323:1239–42. 32091533 10.1001/jama.2020.2648

[4] Guan WJ Liang WH Zhao Y. Comorbidity and its impact on 1590 patients with COVID-19 in China: a nationwide analysis. Eur Respir J 2020;55:10.1183/13993003.00547-2020 PMC7098485 32217650

[5] Alqahtani JS Oyelade T Aldhahir AM. Prevalence, severity and mortality associated with COPD and smoking in patients with COVID-19: a rapid systematic review and meta-analysis. PLoS One 2020;15:e0233147. 32392262 10.1371/journal.pone.0233147 PMC7213702

[6] Sanchez-Ramirez DC Mackey D. Underlying respiratory diseases, specifically COPD, and smoking are associated with severe COVID-19 outcomes: a systematic review and meta-analysis. Respir Med 2020;171:106096. 32763754 10.1016/j.rmed.2020.106096 PMC7391124

[7] Zhao Q Meng M. The impact of COPD and smoking history on the severity of COVID-19: a systemic review and meta-analysis. J Med Virol 2020;92:1915–21. 32293753 10.1002/jmv.25889 PMC7262275

[8] Alberca RW Lima JC de Oliveira EA. COVID-19 disease course in former smokers, smokers and COPD patients. Front Physiol 2020;11:637627. 33584342 10.3389/fphys.2020.637627 PMC7873569

[9] Attaway AA Zein J Hatipoğlu US. SARS-CoV-2 infection in the COPD population is associated with increased healthcare utilization: an analysis of Cleveland clinic's COVID-19 registry. EClinicalMedicine 2020;26:100515. 32869011 10.1016/j.eclinm.2020.100515 PMC7449663

[10] Graziani D Soriano JB Del Rio-Bermudez C. Characteristics and prognosis of COVID-19 in patients with COPD. J Clin Med 2020;9: doi: 10.3390/jcm9103259. 10.3390/jcm9103259 PMC7600734 33053774

[11] He Y Xie M Zhao J Liu X. Clinical characteristics and outcomes of patients with severe COVID-19 and chronic obstructive pulmonary disease (COPD). Med Sci Monitor Int Med J Exp Clin Res 2020;26:e927212. 10.12659/MSM.927212 PMC7491229 32883943

[12] Lee SC Son KJ Han CH Park SC Jung JY. Impact of COPD on COVID-19 prognosis: a nationwide population-based study in South Korea. Sci Rep 2021;11:3735. 33580190 10.1038/s41598-021-83226-9 PMC7880985

[13] Pezzuto A Tammaro A Tonini G Ciccozzi M. COPD influences survival in patients affected by COVID-19, comparison between subjects admitted to an internal medicine unit, and subjects admitted to an intensive care unit: an Italian experience. J Med Virol 2021;93:1239–41. 33026657 10.1002/jmv.26585 PMC7675494

[14] World Health Organization interim guidance. Clinical management of severe acute respiratory infection when novel coronavirus (nCoV) infection is suspected: interim guidance. Available at: https://www.who.int/publications-detail/clinical-management-of-severe-acute-respiratory-infection-when-novel-coronavirus-(ncov)-infection-is-suspected. Published: January 28, 2020; Accessed: January 31, 2020.

[15] Zhou F Yu T Du R. Clinical course and risk factors for mortality of adult inpatients with COVID-19 in Wuhan, China: a retrospective cohort study. Lancet (London, England) 2020;395:1054–62. 10.1016/S0140-6736(20)30566-3 PMC7270627 32171076

[16] Bai Y Liang Y Wang F. Clinical course and outcomes of COVID-19 patients with a history of cerebrovascular disease: a retrospective study in Wuhan. Ann Transl Med 2021;9:988. 34277788 10.21037/atm-21-2237 PMC8267260

[17] Matos J Paparo F Mussetto I. Evaluation of novel coronavirus disease (COVID-19) using quantitative lung CT and clinical data: prediction of short-term outcome. Eur Radiol Exp 2020;4:39. 32592118 10.1186/s41747-020-00167-0 PMC7318726

[18] The National Health Commission of People's Republic of China. Interpretation of New Coronavirus Pneumonia Diagnosis and Treatment Plan (Trial Version 6) (in Chinese). Available at: http://www.nhc.gov.cn/yzygj/s7652m/202002/54e1ad5c2aac45c19eb541799bf637e9.shtml. Accessed April 3, 2020.

[19] Thygesen K Alpert JS Jaffe AS. Fourth universal definition of myocardial infarction (2018). Circulation 2018;138:e618–51. 30571511 10.1161/CIR.0000000000000617

[20] EASL clinical practice guidelines: drug-induced liver injury. J Hepatol 2019;70:1222–61. 30926241 10.1016/j.jhep.2019.02.014

[21] Kidney Disease: Improving Global Outcomes (KDIGO) Acute Kidney Injury Work Group. KDIGO clinical practice guideline for Acute Kidney Injury. Kidney Int Suppl 2012;2:01–138. 2012.

[22] Iba T Nisio MD Levy JH Kitamura N Thachil J. New criteria for sepsis-induced coagulopathy (SIC) following the revised sepsis definition: a retrospective analysis of a nationwide survey. BMJ Open 2017;7:e017046. 10.1136/bmjopen-2017-017046 PMC5623518 28963294

[23] Taylor FB Jr Toh CH Hoots WK Wada H Levi M. Towards definition, clinical and laboratory criteria, and a scoring system for disseminated intravascular coagulation. Thromb Haemost 2001;86:1327–30. 11816725

[24] Wang L He W Yu X. Coronavirus disease 2019 in elderly patients: characteristics and prognostic factors based on 4-week follow-up. J Infect 2020;80:639–45. 32240670 10.1016/j.jinf.2020.03.019 PMC7118526

[25] Wendon J Cordoba J Dhawan A. EASL Clinical Practical Guidelines on the management of acute (fulminant) liver failure. J Hepatol 2017;66:1047–81. 28417882 10.1016/j.jhep.2016.12.003

[26] Ranieri VM Rubenfeld GD Thompson BT. Acute respiratory distress syndrome: the Berlin Definition. JAMA 2012;307:2526–33. 22797452 10.1001/jama.2012.5669

[27] Marshall JC Cook DJ Christou NV Bernard GR Sprung CL Sibbald WJ. Multiple organ dysfunction score: a reliable descriptor of a complex clinical outcome. Crit Care Med 1995;23:1638–52. 7587228 10.1097/00003246-199510000-00007

[28] Rhodes A Evans LE Alhazzani W. Surviving sepsis campaign: international guidelines for management of sepsis and septic shock: 2016. Crit Care Med 2017;45:486–552. 28098591 10.1097/CCM.0000000000002255

[29] Liu J Liu Z Jiang W. Clinical predictors of COVID-19 disease progression and death: analysis of 214 hospitalised patients from Wuhan, China. Clin Respir J 2021;15:293–309. 33090710 10.1111/crj.13296

[30] Mella Pérez C Anton Santos JM Gómez Antúnez M. Clinical characteristics and prognosis of COPD patients hospitalized with SARS-CoV-2. Int J Chron Obstruct Pulmon Dis 2020;15:3433–45. 33447021 10.2147/COPD.S276692 PMC7801905

[31] Shang Y Liu T Wei Y. Scoring systems for predicting mortality for severe patients with COVID-19. EClinicalMedicine 2020;24:100426. 32766541 10.1016/j.eclinm.2020.100426 PMC7332889

[32] Chen T Wu D Chen H. Clinical characteristics of 113 deceased patients with coronavirus disease 2019: retrospective study. BMJ (Clin Res ed) 2020;368:m1091. 10.1136/bmj.m1091 PMC7190011 32217556

[33] Grasselli G Tonetti T Protti A. Pathophysiology of COVID-19-associated acute respiratory distress syndrome: a multicentre prospective observational study. Lancet Respir Med 2020;8:1201–8. 32861276 10.1016/S2213-2600(20)30370-2 PMC7834127

[34] O’Donnell DE Banzett RB Carrieri-Kohlman V. Pathophysiology of dyspnea in chronic obstructive pulmonary disease: a roundtable. Proc Am Thorac Soc 2007;4:145–68. 17494725 10.1513/pats.200611-159CC

[35] O’Donnell DE Milne KM James MD de Torres JP Neder JA. Dyspnea in COPD: new mechanistic insights and management implications. Adv Ther 2020;37:41–60. 31673990 10.1007/s12325-019-01128-9 PMC6979461

[36] Alfahad AJ Alzaydi MM Aldossary AM. Current views in chronic obstructive pulmonary disease pathogenesis and management. Saudi Pharm J 2021;29:1361–73. 35002373 10.1016/j.jsps.2021.10.008 PMC8720819

[37] Higham A Mathioudakis A. COVID-19 and COPD: a narrative review of the basic science and clinical outcomes. Eur Respir Rev 2020;29: doi: 10.1183/16000617.0199-2020. 10.1183/16000617.0199-2020 PMC7651840 33153991

[38] Leung JM Niikura M. COVID-19 and COPD. Eur Respir J 2020;56: doi: 10.1183/13993003.02108-2020. 10.1183/13993003.02108-2020 PMC7424116 32817205

[39] Vanfleteren L Spruit MA Wouters EFM Franssen FME. Management of chronic obstructive pulmonary disease beyond the lungs. Lancet Respir Med 2016;4:911–24. 27264777 10.1016/S2213-2600(16)00097-7

[40] Decramer M Janssens W Miravitlles M. Chronic obstructive pulmonary disease. Lancet (London, England) 2012;379:1341–51. 10.1016/S0140-6736(11)60968-9 PMC7172377 22314182

[41] Hoffmann M Kleine-Weber H Schroeder S. SARS-CoV-2 cell entry depends on ACE2 and TMPRSS2 and is blocked by a clinically proven protease inhibitor. Cell 2020;181: 271–80.e8. 10.1016/j.cell.2020.02.052 PMC7102627 32142651

[42] Donoghue M Hsieh F Baronas E. A novel angiotensin-converting enzyme-related carboxypeptidase (ACE2) converts angiotensin I to angiotensin 1-9. Circ Res 2000;87: E1-9. 10.1161/01.res.87.5.e1 10969042

[43] Bai Y Wang E Zhao S. Implications of laboratory tests in disease grading and death risk stratification of COVID-19: a retrospective study in Wuhan, China. Front Med 2021;8:629296. 10.3389/fmed.2021.629296 PMC7938237 33693017

[44] Schett G Manger B Simon D. COVID-19 revisiting inflammatory pathways of arthritis. Nat Rev Rheumatol 2020;16:465–70. 32561873 10.1038/s41584-020-0451-z PMC7304381

[45] Jacobs M Van Eeckhoutte HP Wijnant SRA. Increased expression of ACE2, the SARS-CoV-2 entry receptor, in alveolar and bronchial epithelium of smokers and COPD subjects. Eur Respir J 2020;56:10.1183/13993003.02378-2020 PMC7366177 32675207

[46] Tavares CAM Avelino-Silva TJ. ACE2 expression and risk factors for COVID-19 severity in patients with advanced age. Arq Bras Cardiol 2020;115:701–7. 33111872 10.36660/abc.20200487 PMC8386971

[47] Sungnak W Huang N Bécavin C. SARS-CoV-2 entry factors are highly expressed in nasal epithelial cells together with innate immune genes. Nat Med 2020;26:681–7. 32327758 10.1038/s41591-020-0868-6 PMC8637938

[48] Opal SM Girard TD Ely EW. The immunopathogenesis of sepsis in elderly patients. Clin Infect Dis 2005;41: (Suppl 7): S504–12. 10.1086/432007 16237654

